# A descriptive analysis of human milk dispensed by the Leipzig Donor Human Milk Bank for neonates between 2012 and 2019

**DOI:** 10.3389/fnut.2023.1233109

**Published:** 2023-11-15

**Authors:** Linda P. Siziba, Caroline Baier, Elisabeth Pütz, Rudolf Ascherl, Thomas Wendt, Ulrich H. Thome, Corinna Gebauer, Jon Genuneit

**Affiliations:** ^1^Pediatric Epidemiology, Department of Pediatrics, Medical Faculty, Leipzig University, Leipzig, Germany; ^2^Division of Neonatology, Department of Pediatrics, University of Leipzig Medical Centre, Leipzig, Germany; ^3^Data Integration Centre, University of Leipzig Medical Centre, Leipzig, Germany; ^4^German Center for Child and Youth Health (DZKJ), Leipzig, Germany

**Keywords:** donor human milk (DHM), mother’s own milk (MOM), infant feeding patterns, raw DHM, Leipzig Donor Human Milk Bank (LMB)

## Abstract

**Background:**

Human milk banking has become an important aspect of Nutritional medicine. It is not just about the provision of mother’s own milk (MOM) or donor human milk (DHM) in the hospital, but also a strategy to encourage breastfeeding in the clinical setting and beyond.

**Objective:**

To describe the feeding patterns of hospitalised infants including human milk dispensed by the Leipzig Donor Human Milk Bank (LMB).

**Design:**

A descriptive analysis of daily data on milk feeds dispensed by LMB for hospitalised infants distinguishing between MOM or DHM, either fresh or frozen, and raw/pasteurised milk from 2012–2019.

**Results:**

We included 2,562 infants with median hospitalisation of 23 days, for whom human milk was dispensed on median 76% of those days and other nutrition on the remaining days. Raw MOM and raw DHM comprised 52% and 8% of the dispensed milk, respectively. Dispensing exclusive DHM instead of MOM for at least one full day was required for 55% of the infants, mostly at the beginning but also later during hospitalisation. Exclusive raw DHM was dispensed on at least 1 day for 37% of the infants, in different birthweight strata <1,000 g: 10%, 1,000-1500 g: 11%, 1,500-2500 g: 13% and > 2,500 g: 3%. At discharge, MOM was dispensed for more than 60% of the infants.

**Conclusion:**

During an infant’s hospital stay, LMB dispenses various human milk feeds with interspersed DHM resulting in complex intra-individual and time-variant feeding patterns. LMB dispenses raw MOM and especially raw DHM with the intention to retain the properties of human milk unlike a diet containing pasteurised DHM and/or formula. Although raw DHM comprises a small percentage of all dispensed milk, raw DHM is dispensed for a substantial portion of infants. Our results document that dispensing raw DHM, is possible in routine settings.

## 1. Introduction

Exclusive breastfeeding remains the recommended source of early life nutrition (1). When mother’s own milk (MOM) is not available, donor human milk (DHM) from human milk banks is the recommended alternative (2–4). The number of human milk banks is increasing worldwide (5). Yet, knowledge about actual DHM use in neonatal intensive care units (NICU) is limited (4, 6, 7).

During hospitalisation, infants receive MOM, DHM and formula, exclusively or concurrently, depending on parental consent and availability. Several studies comparing DHM vs. formula have found considerable reductions in the incidence of necrotizing enterocolitis (NEC) (8–10). Head-to-head comparison of DHM to MOM is rarely reported for infant health outcomes such as NEC, growth, mortality and others; most studies (11–14), combine MOM and DHM into a single measure of human milk feeding. Information about the actual percentage contributions of MOM and DHM across the overall hospital stay would be important because MOM is be prioritised when DHM is available, and when pursuing strategies to improve human milk supply (15).

Moreover, little is known about the intra-individual feeding pattern during hospitalisation. However, we are only aware of one recent study (16) that applied unsupervised machine learning to cluster different groups of hospitalised infants by nutritional patterns and other characteristics. Yet, understanding nutritional course and clustering is of great value for insight into the diversity within common clinical profiles or certain treatment practices (4, 16). Notably, the availability of DHM reduces the time with initial parenteral nutrition (10) but the subsequent course of nutrition is underexplored.

Furthermore, DHM is collected and distributed according to standard operating procedures and is generally pasteurised (5, 17, 18). However, some human milk banks in Germany and also in Norway provide both raw and pasteurised DHM used in accordance with local clinical judgement and health of both recipient and donor (19). The Leipzig Donor Human Milk Bank (LMB) is one of the oldest and largest in Germany providing raw and pasteurised, as well as, fresh and frozen DHM and MOM. Therefore, we sought to describe (i) the variety of intra-individual patterns of dispensed milk and (ii) the output of LMB between 2012 and 2019.

## 2. Methods

### 2.1. Data sources and study variables

**Figure 1:**
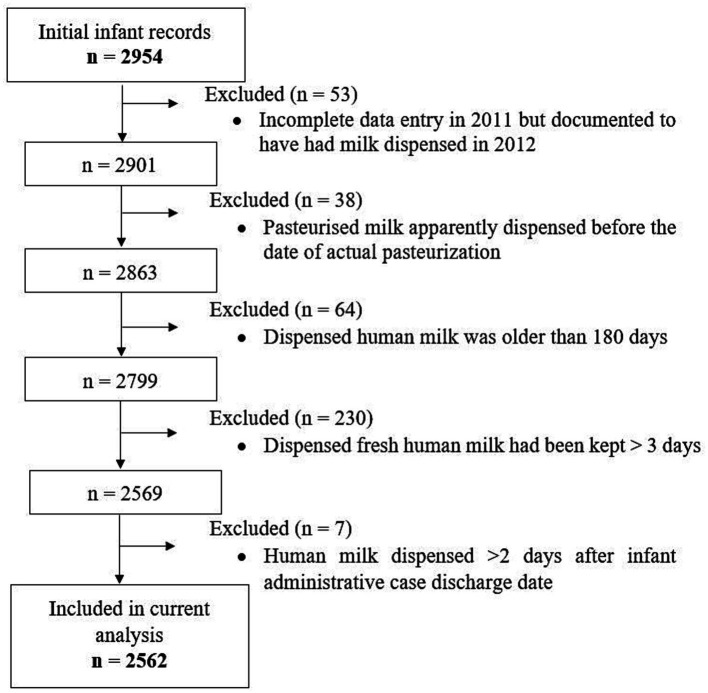
Flowchart of implausible values and observations that were excluded as well as hospitalised infants whose records on dispensed milk types were used for the current analysis.

The Leipzig Donor Human Milk bank (LMB) of Leipzig University Medical Center seamlessly distributes human milk, both MOM and DHM for infants in need especially preterm infants admitted within the Neonatal Intensive Care Unit (NICU) and outside the NICU on other children’s wards in the clinic. Paper-based logs of human milk dispensed daily from LMB date back to at least 2008. Up to now, two medical students under close supervision digitized data from 01/2012 and up to 12/2019 manually into a Microsoft Access database. Although double data entry was not done, visual data checking was done to identify unlikely values, which comprised observations excluded from the current analysis (Figure 1). For this, data extracts with the respective patient identifiers and variables in question were generated and output from SAS statistical software following calculation of the difference between specific dates. For instance, the difference between the date on which milk was pasteurised and the date on which milk was dispensed was calculated. Observations with an unlikely value were assigned a code and these extracts were used to verify the values on the paper documentation. Those with typing errors were corrected, but if values were implausible, these were excluded. The Ethics board of Leipzig University granted Ethical Approval.

Records included the date when human milk was dispensed, patient identifiers, the specific types of human milk dispensed on each day (i.e., raw and pasteurised, as well as, fresh and frozen MOM or DHM) and the date of pasteurization if applicable. Of note, fresh milk was kept at 4°C for a maximum of 3 days, after which it was frozen and stored at −20°C for a maximum of 6 months. DHM was pasteurised if the donor was positive for cytomegalovirus infection (CMV) and/or their skin swab for coagulase-negative Staphylococci colony-forming unit (CFU) was >10^4^/ml. DHM was discarded if any Enterobacteriaceae were detected. MOM was pasteurised for infants <28.0 gestational weeks, for CMV-positive mothers up to 32.0 gestational weeks, and/or if MOM contained >10^5^/ml total bacterial count. Pasteurisation was also dependent on how long the milk would had sat before or until it arrived at LMB after being pumped/expressed by the donor or the mother. Raw milk (MOM or DHM) refers to non-heat treated milk, kept chilled or frozen in the refrigerator or freezer, respectively. Availability of frozen milk was also dependent on milk intake, more milk at long intervals from dispensing, which translates to more milk in the freezer. There was neither information on actual milk volume, biological composition of MOM or DHM, nor on fortification. Further, we had no documentation of actual milk fed to the infant but used daily dispensed human milk as a proxy for intra-individual feeding patterns, which may be prone to some error, e.g., if clinically required nutrition changed during the day.

Data for the current analysis were coded to represent eight different types of human milk: fresh raw MOM (rMOM), frozen rMOM, fresh pasteurised MOM (pMOM), frozen pMOM, fresh raw DHM (rDHM), frozen rDHM, fresh pasteurised DHM (pDHM), and frozen pDHM. These data were merged with information on administrative data, admission and discharge dates, age at admission, and gestational age obtained from hospital records by our hospital’s Data Integration Center. Days during hospitalisation on which the infant had no milk dispensed from LMB were coded as “other nutrition” comprising other forms of parenteral and/or enteral nutrition (e.g., specialized/created diets, infant formula, days when an infant was breastfed). Initially, data from both sources were available from 2,954 infants (Figure 1). We finally included 2,684 administrative cases belonging to 2,562 infants; *n* = 90, *n* = 13 and *n* = 2 infants were admitted twice, three and four times, respectively, and we merged their administrative cases.

### 2.2. Statistical analysis

During the infant’s hospitalisation, each day comprised exclusively one, a mix of the eight human milk types mentioned above, or – if no feeds were dispensed – other nutrition. A modified lasagna plot was used to depict the intra-individual feeding patterns up to the first 100 days of hospitalisation for a subset of infants born and admitted on the same day in 2019 and hospitalised for at least 7 days. For subsequent analyses, milk types on mixed days were weighted by the inverse of the count of distinct milk types on the given day. For each infant, relative proportions were calculated for each milk type by sum of the weighted days divided by the overall number of days for which human milk was dispensed, i.e., percentage contributions of distinct milk types to the overall milk dispensed during hospitalisation. Summation of distinct milk types was used for aggregation to a higher level, e.g., MOM and DHM without discerning raw/pasteurised or fresh/frozen. A kernel plot was used to visualise the distribution of aggregated MOM and DHM percentage contributions for each infant across all calendar years. The contribution of the milk type covered patient days as a percentage of the summed patient days (i.e., days on which milk was dispensed) per calendar year was visualised in a stacked bar chart. Due to the compositional nature of the percentages of the eight milk types per infant, the data were transformed to *centered log ratios* (20) prior to the Cochran-Armitage test for trend across calendar years. All statistical analyses were done using R (version 3.5.1; R Foundation for Statistical Computing, Vienna, Austria) and SAS (version 9.4, The SAS Institute, Cary, NC, United States).

## 3. Results

### 3.1. Intra-individual patterns of dispensed human milk during the first 100 days of hospitalisation in 2019

**Figure 2:**
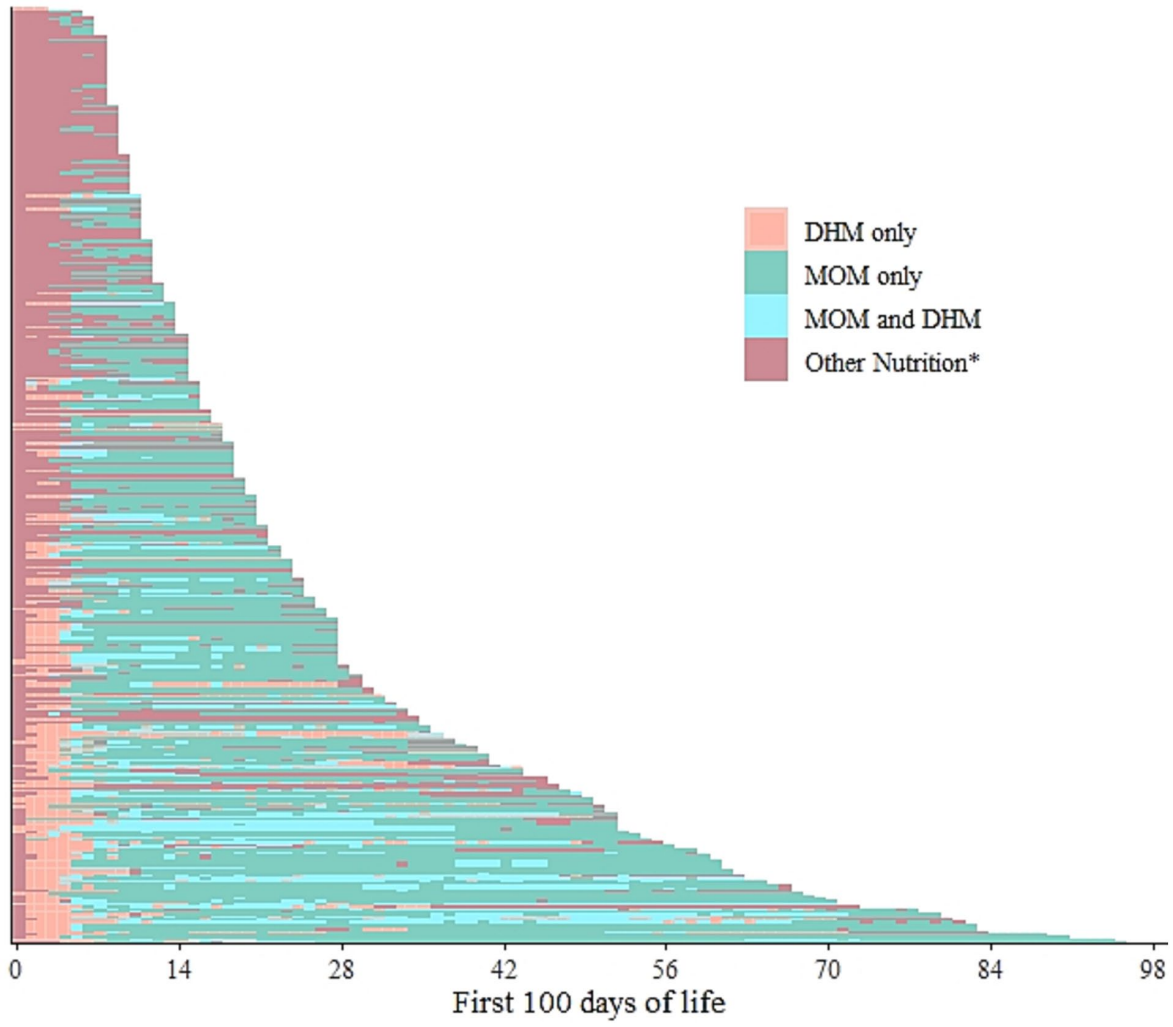
Intra-individual feeding patterns, discerning mother’s own milk (MOM) and donor human milk (DHM), among the infants admitted on the day of birth in 2019 (*n* = 351). The first 100 days of life is equivalent to the first 100 days of hospitalisation. Each line represents one infant. ^*^Other nutrition denotes any other form of either parental/enteral feeds that could have included but not limited infant formula, prescribed nutrition, or even fed directly from the breast.

The 2019 subset comprised n = 351 infants admitted on the day of birth *– or –* within 24 h after birth and hospitalised for at least 7 days, included *n* = 162 girls and *n* = 182 boys, with an average birthweight and gestational age of 2257.9 g [Q1 = 1,550 g, Q3 = 2,900 g] and 34 weeks [Q1 = 31, Q3 = 37], respectively. A modified lasagna plot (Figure 2), each line representing an individual infant, depicts a variety of intra-individual patterns based on dispensed human milk during hospitalisation, though some common features appeared. Other nutrition was the predominant initial form of nutrition, followed by DHM especially for those with a longer hospital stay, and subsequently followed by MOM, which was then supplied for a longer period. There was also a plethora of instances in which DHM exclusively or a mix of DHM and MOM (i.e., separately dispensed on the same day) was interspersed between days of pure MOM; depicting the successful substitution of DHM for MOM to avoid the use of infant formula. Other nutrition was the only form of nutrition for the entire hospitalisation period for some infants.

**Figure 3:**
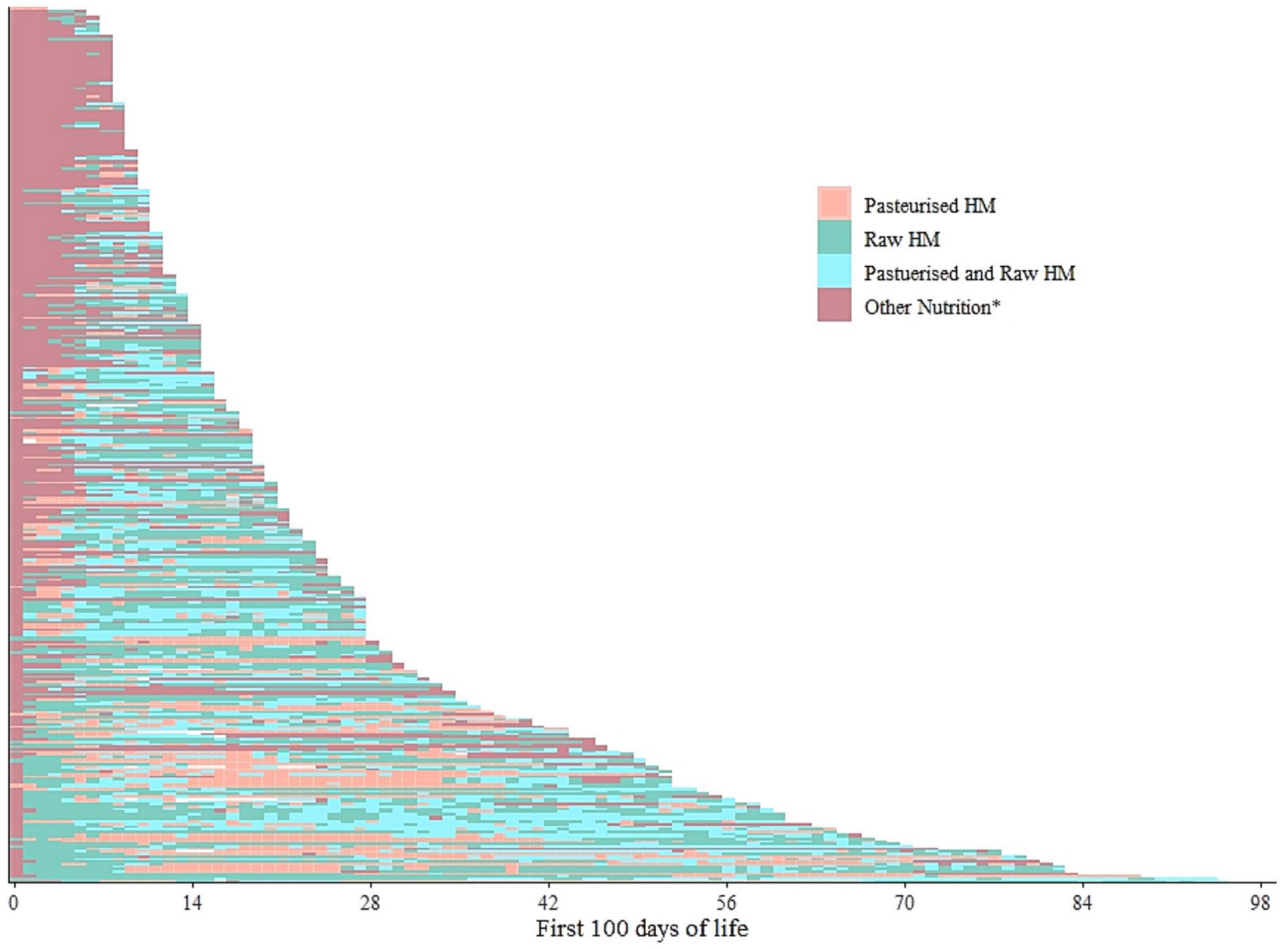
Intra-individual feeding patterns, discerning raw and pasteurised human milk, among the infants admitted on the day of birth in 2019 (*n* = 351). The first 100 days of life is equivalent to the first 100 days of hospitalisation. Each line represents one infant. HM- Human milk. *Other nutrition denotes any other form of either parental/enteral feeds that could have included but not limited infant formula, prescribed nutrition, or even fed directly from the breast. Pasteurized and raw HM refer to the respective milks dispensed on the same day but not mixed.

We further categorised the dispensed human milk into raw or pasteurised, disregarding the MOM or DHM category. The resulting modified lasagna plot (Figure 3) showed slightly more complex intra-individual patterns among the same *n* = 351 infants. Initial human milk seemed to be more often raw, i.e., raw DHM (Supplementary Figure S1) and the graph suggested a higher degree of interspersed patterns while dispensing a mix of both raw and pasteurised milk (i.e., separately dispensed on the same day) on the same day may have occurred more often with longer hospitalisation.

### 3.2. Infants for whom LMB dispensed human milk between 2012 and 2019

In total, LMB dispensed at least one human milk feed for 2,562 hospitalised infants between 2012 and 2019: 2030 singletons, 237 pairs of twins, 18 sets of triplets and 1 set of quadruplets. Almost 90% (*n* = 2,273) of the infants were admitted on the same day they were born; the median age at admission of the remainder was 10 days (Q1 = 2, Q3 = 36, Supplementary Table S1). The median duration of hospitalisation was 23 days [Q1 = 14, Q3 = 41; total of 84,991 days]. On average 70% of the hospitalization, days (62,715 patient-days) were covered with dispensed human milk (i.e., any MOM or any DHM). Other nutrition was the form of nutrition on the remainder of the days (i.e., 30%). For most infants (90%), exclusive MOM was dispensed on at least 1 day, even within the different gestational age strata (Supplementary Table S1), and on average almost 50% of the days of hospitalisation (Table 1); at discharge, MOM was dispensed for 60% of the infants (Supplementary Table S1). Dispensing exclusive raw DHM for at least 1 day, presumably due to lack of raw MOM, was required in more than a third (37%, *n* = 955, Supplementary Table S1) of the infants, in the different birthweight strata (Supplementary Table S2) and on average on 5% of the days of hospitalisation (Table 1). Exclusive fresh raw MOM, i.e., the nutritional gold standard, was dispensed on average on 21% of the days of hospitalisation (Table 1).

**Table 1:** Average (arithmetic mean and median) percentages of hospitalised days per infant on which exclusive and mixed human milk feeds were dispensed between 2012 and 2019.

Dispensed human milk feed	Mean (sd)	Median [Q1, Q3]
Any human milk was dispensed	70 (27)	76 [50, 94]
Exclusive MOM was dispensed	48 (29)	50 [20, 72]
Exclusive DHM was dispensed	13 (19)	5 [0, 17]
Mixed MOM and DHM were dispensed	9 (18)	0 [0, 9]
Exclusive fresh milk was dispensed	41 (26)	43 [17, 63]
Exclusive fresh unpasteurised milk was dispensed	22 (20)	16 [4, 35]
Exclusive fresh unpasteurised MOM was dispensed	21 (20)	15 [3, 35]
Exclusive unpasteurised DHM was dispensed	5 (10)	0 [0, 6]

MOM, Mother’s own milk; DHM, Donor human milk; Q1, First quartile; Q3, Third quartile.

**Figure 4:**
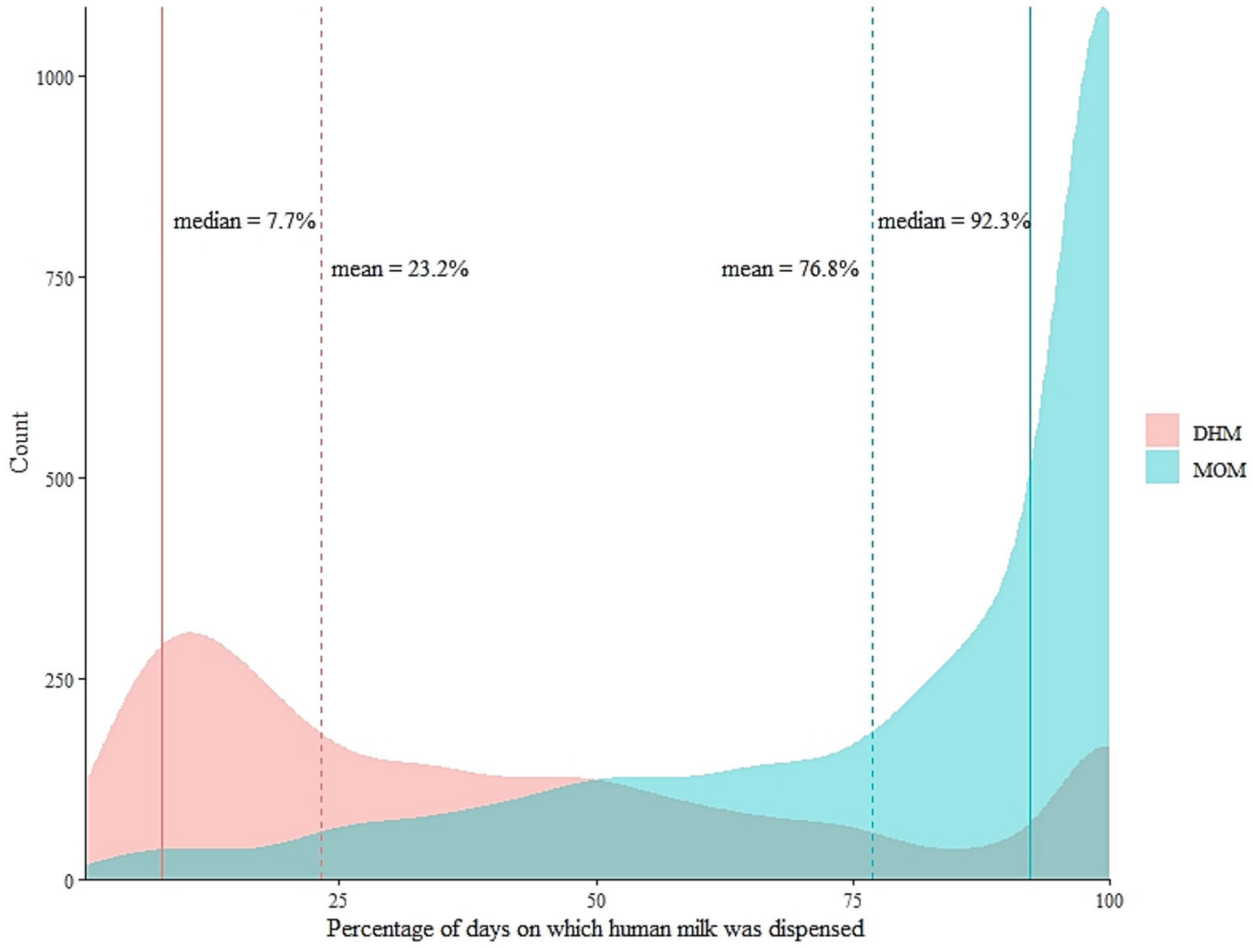
The distribution of mother’s own milk (MOM) and donor human milk (DHM) as percentages of the total number of days on which any human milk was dispensed for each infant between the years 2012 and 2019. Dotted lines show the arithmetic mean of the percentage contributions of the respective human milk type. *N* = 859 infants fall below the means of MOM, *n* = 1703 fall below the means of DHM.

For the subsequent analyses, we rescaled the denominator for each infant by omitting days with other nutrition to the total number of days during which any human milk was dispensed. The percentage contributions of MOM and DHM were displayed in a density plot by overlay to emphasize the different distribution shapes (Figure 4). The overlay therefore does not imply that LMB dispensed a mix of MOM and DHM for a given infant. On the right margin of the graph, 100% of the days were covered with dispensed DHM or MOM for *n* = 166 and *n* = 1,087 infants, respectively. For the remainder of the infants (*n* = 1,309), their patient days were covered by a mix of both MOM and DHM (i.e., dispensed separately on the same day). Although there is no clear cut-off, the density of the MOM-curve takes a steeper increase around 75% of the patient days covered with human milk for *n* = 1738 infants. Suggesting there is no clear break in the shape of the distribution for neither MOM nor DHM. That is, is not possible to pick a meaning cut off for DHM because 75% would not split the sub-population. If we were to, then 50% would be a potentially meaningful cut-off thereof, as it offers a potentially even split.

### 3.3. Overall output of LMB

**Figure 5:**
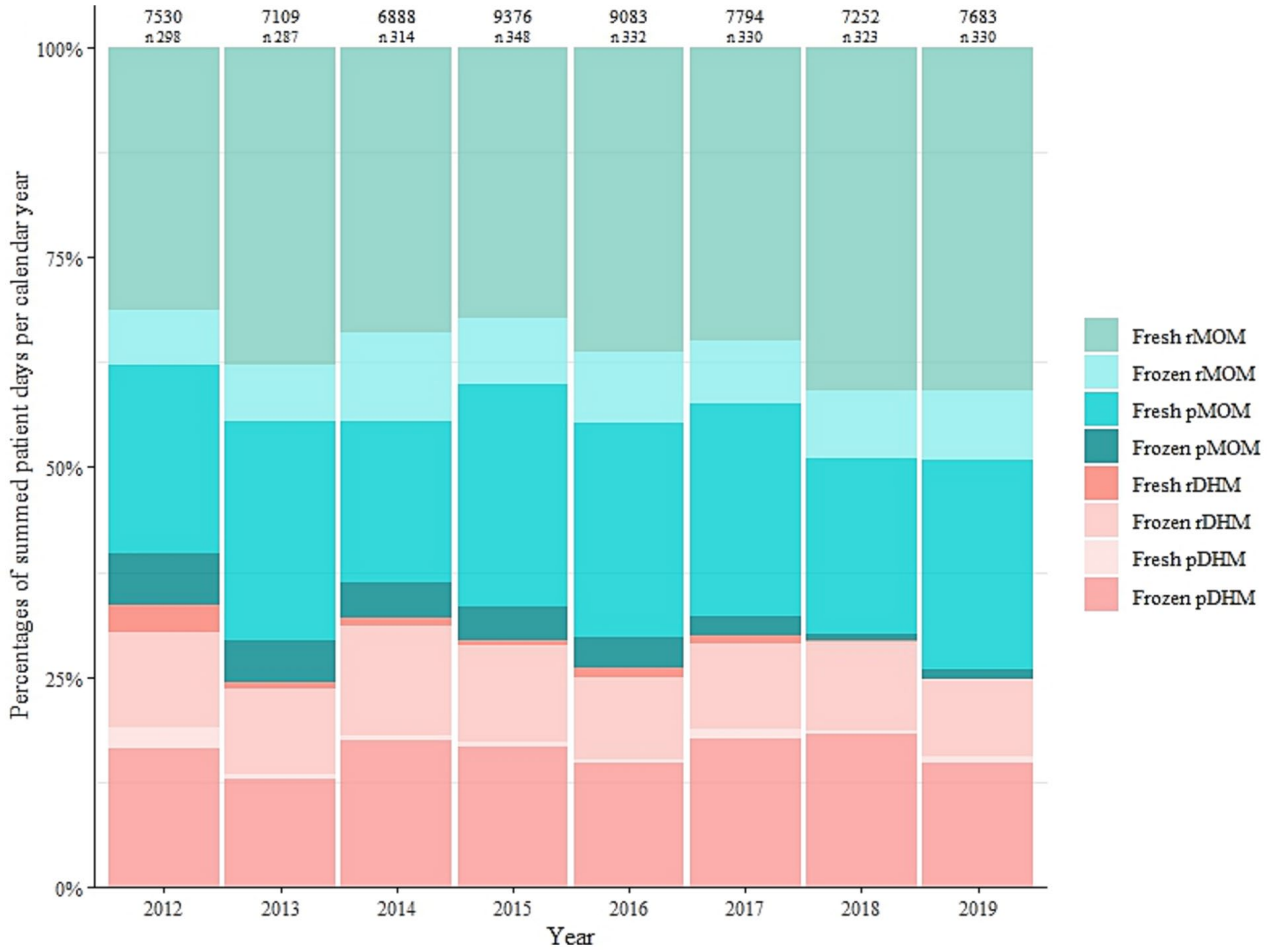
Milk type covered patient days as a percentage of summed patient days per calendar year between 2012 and 2019. Values shown above the plots are the aggregate number of days on which milk was dispensed each year and the number (n) of infants for whom the Leipzig Donor Human Milk Bank (LMB) dispensed milk each year. MOM, mother’s own milk; rMOM, raw mother’s own milk; pMOM, pasteurised mother’s own milk; DHM, donor human milk; rDHM, raw donor human milk; pDHM, pasteurised donor human milk.

Aggregating across all infants, LMB dispensed human milk on 62,715 patient-days (i.e., sum of days on which milk was dispensed) across the eight-year study period (Supplementary Table S1). In each of these years, fresh raw MOM was the most commonly dispensed type (Figure 5; Supplementary Table S3). However, there was borderline statistical significance (p_trend_ = 0.055) for an overall trend in dispensing higher or lower percentages of the milk types. There was a marked drop in the percentages of dispensed fresh raw DHM, frozen raw DHM, and fresh pasteurized DHM from 2012 to 2013 accompanied by an increase in the percentage of fresh raw MOM, which may drive the overall and individual trends (Supplementary Table S4). In a more aggregated view, there were statistically significant trends in dispensing less pasteurised and less frozen milk over the years (*p* < 0.05, Supplementary Table S4). Although the proportions of DHM comprised 8% of overall milk types, raw DHM was dispensed exclusively for 37% of the infants, that is, a substantial portion of infants.

## 4. Discussion

Leipzig Donor Human Milk Bank (LMB) dispensed human milk for 2,562 infants between 2012 and 2019 with a median hospital stay of 23 days, on a median of 76% of those days amounting to 62,715 patient-days covered with human milk. Using daily data on dispensed milk as a proxy for actual infant feeding, we document an explicit variety of intra-individual feeding patterns during the first 100 days of hospitalisation. Moreover, we were able to discern the percentage contribution of MOM and DHM, that is, the proportion of days on which a specific milk type was dispensed for an infant. These data could potentially improve accuracy in grouping infants into distinct human milk feeds accounting for co-exposure to each milk feed in studies contrasting DHM and MOM exposure. DHM was dispensed to potentially bridge the days until MOM was available (21). The supply of raw milk in general has consistently increased over the years, with raw MOM amounting to almost 60% of all dispensed milk in 2019 when raw DHM had a share of 5%. Although this share of DHM was small it was dispensed for a substantial portion of the infants (*n* = 129, 40%).

We identified plausible intra-individual feeding patterns during the first 100 days of hospitalisation. Notably, the time-variant nature of these potential feeding patterns is likely due to milk availability, but also the infant’s clinical state. Although other nutrition was the predominant initial form of nutrition, the ultimate goal is to optimize the use of MOM during early life. In our study, 90% of the infants received exclusive MOM at least once marking a high initiation rate and at discharge, MOM and DHM were dispensed for 60% and 3% of the infants, respectively. However, none of the infants are discharged while dependent on DHM, thus this result should be interpreted with caution, as it may have been subject to documentation error. Compared to nationwide figures (22), all of this suggests that the services of LMB do not impact on breastfeeding rates (23). Similar to other studies (24–27), we show that DHM is dispensed as a bridge until MOM is available, and potentially overcoming initial breastfeeding difficulties. On one hand, other studies reported a decreased use of MOM after the introduction of DHM, despite having received some MOM prior to DHM availability (11, 28). On the other hand, the availability of a human milk bank resulted in a stable use of MOM while the use of infant formula milk in the NICU decreased (29, 30). As such, our results further highlight the importance of a human milk bank in supporting mothers to overcome breastfeeding challenges (24, 31–33).

Describing the complex, time-varying feeding exposures during hospitalisation is an essential first step to ascertain whether the sequence of events supports a causal association of MOM or DHM feeding with the onset of an infant’s clinical outcome. We are aware of a recent study (16) clustering infants admitted to neonatal units in England according to clinical and feeding data. While this study shows time curves of the proportions of infants for whom a nutritional component (e.g., MOM, DHM) was dispensed, it does not provide insight into intra-individual sequences but rather identifies clustered groups of infants with varying percentage contributions of milk types to overall feeding. Most other previous studies largely neglect time-variant aspects of human milk feeding and employ crude categories of milk type proportions over the whole hospital stay.

In light of this, it has been pointed out (11), that previously reported beneficial effects and/or associations using such predefined cut-offs, e.g., 75% of feeds during hospitalisation covered with MOM, may well be driven by co-exposure to MOM and DHM. Our results on intra-individual patterns and on distributional aspects of MOM and DHM co-exposure highlight the importance of choosing cut-offs carefully from the underlying research population and desired contrast, rather than from the “learned presentations” or arbitrary cut-offs. As such, the services of the milk bank are not just about supplying hospitalised infants with DHM, but also about creating a strategy that encourages breastfeeding in the clinical setting with a public health perspective. More research is therefore needed to investigate the dose-and time-dependent associations of the potential feeding patterns identified in this current descriptive analysis with infant health outcomes. Moreover, it is only until recently that the use of DHM has been limited only to vulnerable groups of infants based on gestational age cut-off and birth weight (24). This warrants further research to provide more insight in the use of DHM in populations beyond the NICU (12).

Adhering to strict standards and extensive screening (19, 34, 35), LMB dispensed raw DHM for all infants across different gestational ages and birthweight. Without this, these feeds would have had to be covered by pasteurised DHM. Although the nutritional composition of raw DHM is not thoroughly documented (36), the intention therefore is to retain as much of the active biological and microbial contents within the milk (37). However, this practice is not without challenges, as it requires extensive screening, with potentially large volumes of DHM being discarded due to high microbial counts. Still, the use of raw milk in general and particularly raw DHM is still a unique feature of LMB and some other human milk banks in Germany and few other countries.

Although we show a reduced distribution of DHM over the 8 year study period in Leipzig, the reduction was compensated by increased use of MOM, and not by formula feeding. The German Neonatal Network (GNN) (38) shows an increased use of (pasteurised) DHM for enteral feeding in Germany between 2013 and 2019, especially in very low birth weight infants. Similarly, higher rates of DHM use were reported in the United States (3, 7) and United Kingdom (16). Although conclusions from the GNN are based on cumulative data, hospital practices of providing DHM vary by geographical region and institution. DHM provides an indispensable bridge to successful optimal forms of nutrition, but MOM should be prioritised when pursuing strategies to increase supply and provision of human milk (31, 39).

A limitation of our study is the lack of actual volume of milk supplied and fed to the infant, leaving us with taking dispensed milk weighted by the inverse of the count of different milk types per day as a proxy. We lack data on daily caloric intake and on nutritional and biological composition of either MOM or DHM and donor characteristics. Standard fortification was done during the observed time frame and target fortification was only carried out as part of a clinical study during the observation period. However, these data are not included in the records of the LMB. Important strengths of this study are the insights into eight different milk types, actual relative contributions of MOM and DHM can provide, which allow more accurate grouping of infants according to their milk feeds. This allows the comparison of exclusive milk groups thereby reducing the potential confounding effect of combining both MOM and/or DHM in one single group. We also display high-resolution data on plausible feeding patterns during hospitalisation over a defined period, which demonstrates the time-variant nature of feeding that is likely highly relevant for association studies with infant outcomes. This eliminates a major limitation of including MOM and DHM in single matrices, which could lead to inclusion bias and lower generalisability in observational analyses.

In conclusion, forms of nutrition during hospitalisation vary greatly, with interspersed DHM resulting in complex intra-individual time-variant feeding patterns. Thus, cut-offs utilised for classification into predominant MOM or DHM feeding during hospitalisation in previous studies may not always be applicable in otherwise different infant populations. LMB dispenses raw milk, particularly raw DHM with the intention to preserve the properties of human milk unlike a diet containing pasteurised DHM and/or formula. As such, dispensing raw DHM is possible for a substantial portion of infants.

## Data availability statement

The datasets presented in this article are not readily available due to data protection laws, we may not be able to share the raw data. However, the authors are open to sharing aggregate data (for instance, relative concentrations of the different milk types). Requests to access the datasets should be directed to Jon.Genuneit@medizin.uni-leipzig.de.

## Ethics statement

The studies involving humans were approved by Ethics board of Leipzig University. The studies were conducted in accordance with the local legislation and institutional requirements. Written informed consent for participation was not required from the participants or the participants' legal guardians/next of kin in accordance with the national legislation and institutional requirements.

## Author contributions

LS, CB, EP, CG, and JG: conceptualization. CB, JG, EP, LS, UT, CG, RA, and TW: data acquisition. LS, CB, EP, and JG: analysis. LS, CB, EP, CG, RA, UT, and JG: interpretation. LS and JG: drafting of the manuscript. All authors contributed to the article and approved the submitted version.
