# Supplementary material for: A descriptive analysis of human milk dispensed by the Leipzig Donor Human Milk Bank for neonates between 2012 and 2019
Source: Front Nutr. 2023 Nov 15;10:1233109. doi: 10.3389/fnut.2023.1233109 (PMC10684730; doi:10.3389/fnut.2023.1233109)
Supplement: Supplementary file 1 [file Data_Sheet_1.docx]

Supplementary Material

A descriptive analysis of human milk dispensed by the Leipzig Donor Human Milk Bank for neonates between 2012 and 2019

**Linda P. Siziba^1*^, Caroline Baier^1^, Elisabeth Pütz^1^, Rudolf Ascherl^2^, Thomas Wendt^3^, Ulrich H. Thome^2^, Corinna Gebauer^2^and Jon Genuneit^1,4^**

*** Correspondence:** Corresponding Author: Linda.Siziba@medizin.uni-leipzig.de

# Supplementary Figures and Tables

**
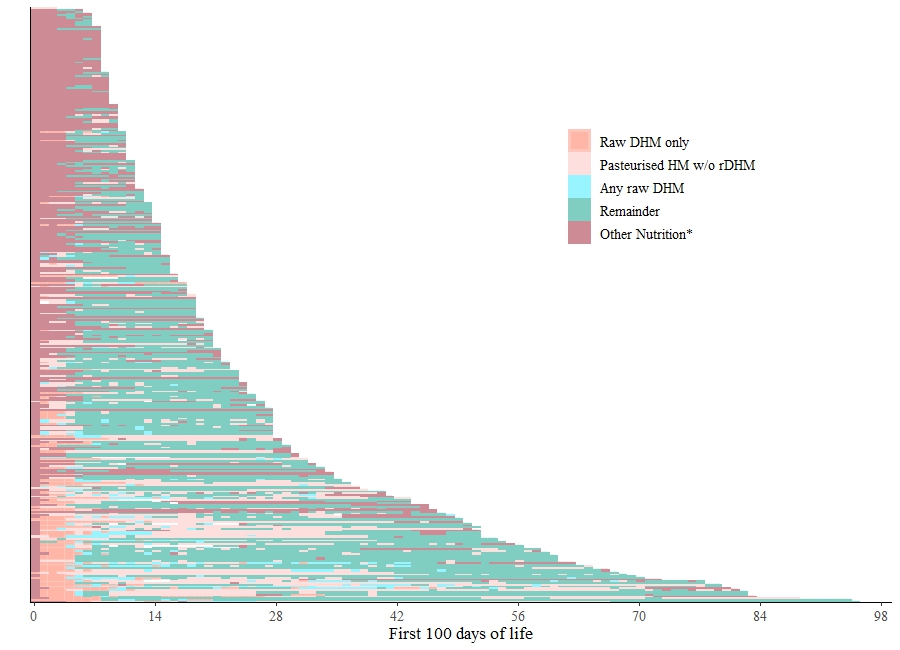
**

**Supplementary Figure S1:** Intra-individual feeding patterns, discerning raw donor human milk (DHM), pasteurised human milk (HM) and other nutrition, among the infants born and admitted on the same day in 2019 (n=351). Each line represents one infant. *Other nutrition: any other form of either parental/enteral feeds that could have included but not limited infant formula, prescribed nutrition, or even fed directly from the breast; w/o: without; rDHM: raw donor human milk. The category pasteurized HM w/o rDHM means that on a given day, only pasteurised HM was dispensed, no raw DHM on that given day was dispensed.

**Table S1:** Output of the Leipzig Milk Bank and admission information of the infants for whom human milk was dispensed between 2012 and 2019 stratified by gestational age

|  | **n (%)** | **GA < 28 weeks** | | **GA 28 ≥and < 32 weeks** | **GA 32** ≥ **and < 37 weeks** | **≥ 37 weeks** |
| --- | --- | --- | --- | --- | --- | --- |
| Cases | 2684 | 233 | | 649 | 993 | 549 |
| Children | 2562 | 231 | | 644 | 987 | 546 |
| Hospital stay (days)^1^ | 27 (37) | 41 (29) | | 54 (56) | 19 (20) | 25 (28) |
| Not admitted at birth^2^ | 289 (11) | 8 (3) | | 18 (3) | 43 (4) | 83 (15) |
| Age at admission (days)^3^ | 10  [2, 36] | 53  [13, 94] | | 10  [1, 22] | 1  [1, 5] | 2  [1, 8] |
| Patient days^4^ | 62715 | 15886 | | 23785 | 14661 | 6051 |
| Children [n (%)] with at least one day covered exclusively with | | |  |  |  |  |
| MOM | 2304 (90) | 207 (90) | | 565 (88) | 881 (89) | 499 (91) |
| DHM | 1405 (55) | 208 (90) | | 590 (92) | 490 (50) | 106 (19) |
| Fresh milk | 2334 (91) | 215 (93) | | 589 (91) | 894 (91) | 500 (92) |
| Fresh raw milk | 2042 (80) | 184 (80) | | 491 (76) | 800 (81) | 444 (81) |
| Fresh raw MOM | 1978 (77) | 161 (70) | | 468 (73) | 786 (80) | 441 (81) |
| raw DHM | 955 (37) | 205 (89) | | 493 (77) | 200 (20) | 51 (9) |
| Children [n (%)] whose milk orders at discharge were exclusive | | |  |  |  |  |
| MOM | 1527 (60) | 119 (52) | | 393 (61) | 654 (66) | 299 (55) |
| DHM | 83 (3) | 11 (5) | | 33 (5) | 27 (3) | 10 (2) |
| MOM and DHM | 94 (4) | 7 (3) | | 32 (5) | 46 (5) | 7 (1) |
| Other | 858 (33) | 94 (41) | | 186 (29) | 260 (26) | 230 (42) |

^1^Mean (SD). ^2^Infants not admitted on the day of their birth. ^3^Median (Interquartile range, IQR). ^4^Patient days were calculated as the sum of all days on which any human milk was dispensed. MOM: Mother’s own milk; DHM: Donor human milk; GA: Gestational Age. Frequencies in the GA columns may not add up to the number in the total column due to missings.

**Table S2:** Output of the Leipzig Milk Bank and admission information of the infants for whom human milk was dispensed between 2012 and 2019 stratified by birthweight

|  | ELBW | VLBW | LBW | Other |
| --- | --- | --- | --- | --- |
|  | **< 1000g** | **1000g ≤ and < 1500g** | **1500g ≤ and < 2500g** | **≥ 2500g** |
| Cases | 277 | 336 | 1092 | 718 |
| Children | 275 (100) | 332 (100) | 1086 (100) | 714 (100) |
| Hospital stay (days)^1^ | 70 (83) | 41 (29) | 29 (34) | 22 (23) |
| Not admitted at birth^2^ | 6 (2) | 11 (3) | 41 (4) | 93 (13) |
| Age at admission (days)^3^ | 47 [12, 88] | 8 [1, 22] | 2 [1, 6] | 2 [1, 6] |
| Total milk days | 18285 | 13286 | 20895 | 7888 |
| Children [n (%)] with at least one day covered exclusively with |  |  |  |  |
| MOM | 247 (90) | 283 (85) | 972 (90) | 649 (91) |
| DHM | 256 (93) | 302 (91) | 672 (62) | 164 (23) |
| Fresh milk | 260 (95) | 297 (89) | 990 (91) | 650 (91) |
| Fresh raw milk | 223 (81) | 237 (71) | 880 (81) | 578 (81) |
| Fresh raw MOM | 198 (72) | 218 (66) | 867 (80) | 572 (80) |
| raw DHM | 250 (91) | 277 (83) | 352 (32) | 70 (10) |
| Children [n (%)] whose milk orders at discharge were exclusive |  |  |  |  |
| MOM | 145 (53) | 189 (57) | 724 (67) | 407 (57) |
| DHM | 12 (4) | 22 (7) | 31 (3) | 16 (2) |
| MOM and DHM | 9 (3) | 19 (6) | 56 (5) | 8 (1) |
| Other | 109 (40) | 102 (31) | 275 (25) | 283 (40) |

^1^Mean (SD). ^2^Infants not admitted on the day of their birth. ^3^Median (Interquartile range, IQR). MOM: Mother’s own milk; DHM: Donor human milk; ELBW: Extremely low birthweight; VLBW: Very low birthweight; LBW: Low birthweight. Frequencies in the birth weight columns may not add up to the number in the total column due to missings.

**Table S3:** Average percentages of each of the eight human milk types dispensed between 2012 and 2019

|  | **2012** | **2013** | **2014** | **2015** | **2016** | **2017** | **2018** | **2019** | **Overall** |
| --- | --- | --- | --- | --- | --- | --- | --- | --- | --- |
| Children (n) | 298 | 287 | 314 | 348 | 332 | 330 | 323 | 330 | 2562 |
| Fresh rMOM | 40 | 47 | 45 | 47 | 46 | 45 | 47 | 54 | 46 |
| Frozen rMOM | 4 | 4 | 6 | 6 | 6 | 5 | 5 | 5 | 5 |
| Fresh pMOM | 22 | 25 | 21 | 23 | 25 | 26 | 22 | 25 | 24 |
| Frozen pMOM | 3.2 | 2.8 | 1.9 | 1.8 | 1.7 | 1.1 | 0.3 | 0.3 | 1.6 |
| Fresh rDHM | 2.3 | 0.3 | 0.4 | 0.2 | 1.1 | 0.3 | 0.0 | 0.1 | 0.6 |
| Frozen rDHM | 9 | 7 | 7 | 7 | 7 | 6 | 9 | 5 | 7 |
| Fresh pDHM | 2.2 | 0.4 | 0.4 | 0.6 | 0.2 | 0.7 | 0.1 | 0.3 | 0.6 |
| Frozen pDHM | 17 | 14 | 17 | 15 | 13 | 15 | 18 | 11 | 15 |

MOM: mother’s own milk; rMOM: raw mother’s own milk; pMOM: pasteurised mother’s own milk; DHM: donor human milk; rDHM: raw donor human milk; pDHM: pasteurised donor human milk. Percentages represent the relative controbution of each milk type to the total number of days on which human milk was dispensed

**Table S4:** Average percentage contributions of aggregate human milk types dispensed between 2012 and 2019

|  | **2012** | **2013** | **2014** | **2015** | **2016** | **2017** | **2018** | **2019** | **Overall** | **Pvalue^1^** | **Pvalue^2^** |
| --- | --- | --- | --- | --- | --- | --- | --- | --- | --- | --- | --- |
| Children (n) | 298 | 287 | 314 | 348 | 332 | 330 | 323 | 330 | 2562 |  |  |
| MOM | 69 | 79 | 75 | 77 | 79 | 78 | 74 | 84 | 77 | 0.7185 | 0.0201 |
| DHM | 31 | 21 | 25 | 23 | 21 | 22 | 26 | 16 | 23 | 0.1365 | 0.2935 |
| rMOM | 44 | 51 | 51 | 53 | 52 | 51 | 52 | 59 | 52 | 0.7310 | 0.4028 |
| rDHM | 12 | 7 | 8 | 7 | 8 | 6 | 9 | 5 | 8 | <.0001 | 0.0013 |
| Raw milk | 56 | 58 | 59 | 60 | 60 | 57 | 60 | 64 | 59 | 0.5779 | 0.4138 |
| Pasteurised milk | 44 | 42 | 41 | 40 | 40 | 43 | 40 | 36 | 41 | 0.0002 | 0.0443 |
| Fresh milk | 66 | 73 | 67 | 71 | 73 | 72 | 69 | 79 | 71 | 0.2177 | 0.8818 |
| Frozen milk | 34 | 27 | 33 | 29 | 27 | 28 | 31 | 21 | 29 | 0.0057 | 0.2006 |

MOM: Mother’s own milk; DHM: Donor human milk. Linear regression of centered log ratio transformed values was used to determine the Pvalue for trend of the respective ordered milk feeds across the years. ^1^P-value for trend including data from the year 2012. ^2^P-value for trend excluding data from the year 2012.
